# Evaluation of Multiplex Nested Polymerase Chain Reaction for Routine Hepatitis C Virus Genotyping in Egyptian Patients

**DOI:** 10.5812/hepatmon.830

**Published:** 2012-04-30

**Authors:** Mohamed Abbas Shemis, Dina Mohamed El-Abd, Dalia Ibrahim Ramadan, Mohamed Ibrahim El-Sayed, Bassem Shenoda Guirgis, Mohamed Ali Saber, Hassan Mohamed El-Said Azzazy

**Affiliations:** 1 Department of Biochemistry, Theodor Bilharz Research Institute, Giza, Egypt; 2 Department of Clinical and Chemical Pathology, Faculty of Medicine, Cairo University, Cairo, Egypt; 3 Department of Biochemistry, Faculty of Medicine, Cairo University, Cairo, Egypt; 4 Yousef Jameel Science and Technology Research Center, the American University in Cairo, Cairo, Egypt; 5 Department of Chemistry, the American University in Cairo, Cairo, Egypt

**Keywords:** Hepatitis C, Multiplex Polymerase Chain Reaction, Branched DNA Signal Amplification, Assay

## Abstract

**Background:**

At least six HCV (hepatitis C virus) genotypes are unequally distributed worldwide. HCV genotyping guides the selection of treatment regimens and provides important epidemiological markers that enable the outbreak source to be traced and the spread of disease to be controlled. In Egypt, there is an increasing need for cost-effective, fast, and easily performable HCV genotyping assays.Recently, a multiplex PCR assay was developed to determine HCV genotypes. It employs genotype-specific primers, based on sequences of the entire core region and part of the 5’UTR of the genome.

**Objectives:**

In this study, we compared a simple, new, modified multiplex PCR system for HCV genotyping with a commercially available line probe assay (INNO-LiPA) that is based on reverse hybridization.

**Patients and Methods:**

Serum samples from chronic HCV Egyptian patients (n = 73) were genotyped using the modified multiplex PCR assay, and genotypes were verified using the INNO-LiPA HCV II assay.

**Results:**

The modified multiplex PCR method was able to type HCV-4 in 65 of 70 typeable samples (92.86%) and had 100% concordance with the INNO-LiPA assay.

**Conclusions:**

Genotype 4 was the most prevalent genotype in our study. Based on our results, the modified multiplex nested PCR assay is a sensitive and inexpensive alternative for HCV genotyping and can be used in routine diagnostic laboratories. INNO-LiPA may be useful as a second-line assay for genotyping samples that are indeterminate by multiplex PCR. This approach will effect better treatment optimization and a reduction of the spread of HCV.

## 1. Background

HCV genotype 4 (HCV-4) is responsible for more than 80% of HCV infections in the Middle East and Africa and has recently spread to several European countries [1][2]. Egypt has one of the highest rates of HCV worldwide (~15%) and the highest prevalence of HCV-4, which is responsible for nearly 90% of all infections [1][3][4]. Although phylogenetic analysis of a coding region, or even the complete genome, is considered to be the gold standard for identifying HCV genotypes and subtypes [5], this approach is impractical for large-scale genotyping projects, since it is both expensive and time-consuming [6][7]. Thus, a variety of surrogate HCV typing procedures have been developed over the past 10 years, based primarily on ampliﬁcation of viral sequences by PCR. For all of these assays, only one region (eg, the 5’UTR, core) is analyzed as a representative of the entire genome [8][9][10][11][12][13]. Examples of such assays include those developed by Okamoto et al. and Ohno et al. [10][11]. One limitation of the assay by Okamoto et al. (1992) is that it was designed to detect genotypes 1, 2, and 3a [10]. Thus, this system fails to type Egyptian strains, since no genotype 4-specific primers exist [14]. Furthermore, the system also displays a higher number of mixed-infection designations, probably due to non-specific priming [11][15].

A second example is the assay developed by Ohno et al. (1997) [11]. However, it also needs to be revised or updated to enable new genotypesto be identified. In addition, the number of samples of genotypes 3, 4, 5, and 6 that were tested was very small and may not have been sufficient to allow definitive conclusions to be drawn about this method. Moreover, the region that was used to design outer primers might not be suitable if all common subtypes are to be detected with great sensitivity [15]. In an attempt to overcome this limitation and increase the sensitivity of the assay, Idrees (2008) developed an assay that amplifies a region from the 5’UTR, along with the entire core region, using genotype-specific primers [15]. Since the primers were designed based on the nucleotide sequences of many genotypes/subgenotypes—namely, 1a, 1b, 1c, 2a, 2b, 2c, 3a, 3b, 3c, 4a–h, 5a, and 6a HCV isolates—this revised system has much broader applications.

## 2. Objectives

The main goal of this study was to evaluate a modified multiplex PCR system, based on that described by Idrees (2008), for use as a reliable and economical HCV genotyping method for Egyptian patients and to compare the results of this system with those obtained using a commercially available method that is based on the line probe assay, INNO-LiPA HCV II.

## 3. Materials and Methods

### 3.1. Materials

This study was conducted between January 2009 and October 2009 and included 100 anti-HCV-positive serum samples collected from patients who were chronically infected with HCV. Anti-HCV was tested using a third-generation enzyme immunoassay (EIA) (Murex Anti-HCV (Version 4) ABBOTT Diagnostic Division, Murex Biotech S.A. (Pty) Ltd, Kyalami Boulevard, and Republic of South Africa). Samples were taken from the Biochemistry Department of the Theodor Bilharz Research Institute and the Chemical Pathology Department of the Kasr Al-Aini Faculty of Medicine, Cairo University. The study was approved by the ethics committees from both hospitals.

### 3.2. HCV RNA Extraction and PCR Detection

HCV RNA was extracted from 140 μL serum using the QIAamp Viral RNA Mini Kit (QIAgen, Hilden, Germany) and re-suspended in 60 µL buffer. Strict measures were taken throughout the sampling, extraction, and PCR to prevent nucleic acid carryover [16]. Nested reverse-transcription PCR (RT-PCR) was conducted, and HCV RNA-positive samples were genotyped.

### 3.3. HCV Genotyping Using a Modified Multiplex PCR Protocol

3.3.1. cDNA Synthesis and First-Round PCR Amplification

In this study, we modified the multiplex PCR protocol reported by Idrees [15]. The RT and first-round PCR were performed in a single step. Briefly, cDNA synthesis and first-round PCR amplification were performed in a 50 µL reaction volume, containing 20 µL RNA, 50 pmol of each of the universal outer forward and reverse primers (Table 1), 200 µM of each deoxynucleotide (dNTP), 10 U of avian myeloblastosis virus reverse transcriptase (AMV RT) (Promega Madison, WI, USA), 2.5 U of Taq DNA polymerase (Promega Madison, WI, USA), 40 U of RNAsin (Promega Madison, WI, USA), 20 mM Tris-HCl (pH 8.4), and 50 mM KCl. Reactions were performed in a PTC 200 thermal cycler (MJ Research, Watertown, Mass., USA), programmed as follows:42°C for 30 min; 95°C for 5 min; 40 cycles of 94°C for 1 min, 45°C for 1 min, and 72°C for 1 minute; and 72°C for 5 min. This process amplified a 470 bp band, which comprised part of the 5’UTR and the entire core region.

**Table 1:** Universal and Genotype Specific Primers Used for Modified Multiplex Nested PCR

**PCR rounds**	**Sequence a**	**Specificity**	**Polarity**
RT and first PCR	TTG TGG TAC TGC CTG ATA GGG	Universal outer	Sense
	GGA TGT ACC CCA TGA GG(A) TCG	Universal outer	Anti-sense
Second PCR (Mix A)	GTG CCC CGG GAG GTC TCG TAG	Universal inner	Sense
	ACT CCA CCA ACG ATC TGA CC	Type 1a	Anti-sense
	AGC CTT GGG GAT AGG TTG TC	Type 1b	Anti-sense
	CTT ACC CAA ATT GCG TGA CC	Type 1c	Anti-sense
	ACT CCA CCA ACG ATC TGT CC	Type 3a	Anti-sense
	GTG ACC GCT CGG AAG TCT TA	Type 3c	Anti-sense
	CCG TAA AGA GGC CAT GGA TA	Type 4	Anti-sense
Second PCR (Mix B)	GTG CCC CGG GAG GTC TCG TAG	Universal inner	Sense
	CTC CGA AGT CTT CCT TGT CG	Type 2a	Anti-sense
	AGC AAG TAA ACT CCG CCA AC	Type 2b	Anti-sense
	ACC GTT CGG AAG TTT TCC TC	Type 2c	Anti-sense
	AGC CTT GGG GAT AAG GTG AC	Type 3b	Anti-sense
	AAT CCG CAC GTT AGG GTA TG	Type 5a	Anti-sense
	CAG CCT TCG CTT CCA TAA AG	Type 6a	Anti-sense

^a^ Nucleotide inside parentheses is a degenerate nucleotide

3.3.2. Second-Round PCR

**Figure 1:**
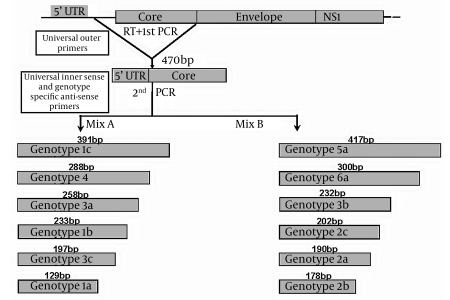
Schematic Design of the Modified Multiplex PCR Assay for HCV Genotyping

Second-Round PCR was performed as in Idrees [15], in which 2 parallel second-round PCR reactions (20 µL each) were conducted for each sample, using the first-round PCR amplicon and primer mixes A or B. Table 1 summarizes the universal and genotype-specific primers. Tubes were placed into a thermocycler that was programmed for 35 cycles as follows: 15 cycles (94°C, 50°C, and 72°C, for 45 s, 45 s, and 1 min respectively); 20 cycles (94°C, 58°C, and 72°C, for 45 s, 45 s, and 1 min, respectively); and a final extension at 72°C for 10 min. Amplicons were then resolved by agarose gel electrophoresis (2.5%), yielding genotype-specific band sizes, which were compared with a 50 bp DNA ladder. The choice of primer combinations (mixes A and B) was decided, based on differences in genotype-specific band sizes. Figure 1 illustrates the multiplex PCR design.

### 3.4. INNO-LiPA

All samples were also analyzed by VERSANT HCV Genotype assay (INNO-LiPA HCV II; Bayer Health Care, Eragny, France) per the manufacturer’s instructions [17].

### 3.5. Statistical Analysis

Data were analyzed using the SPSS, release 17.0. Categorical variables were expressed as rates (%). Correlation and kappa test of agreement between the two assays were performed. P values ≤ 0.05 were considered statistically significant.

## 4. Results

**Figure 2:**
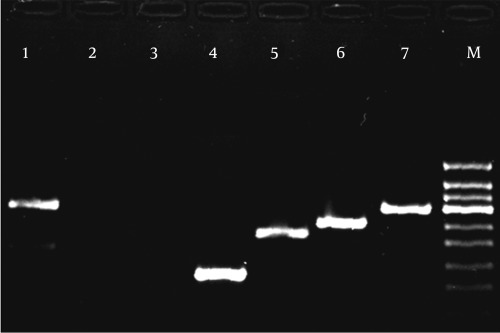
Agarose Gel Electrophoresis of the Modified Multiplex PCR Genotyping Method Showing Genotype-Specific Bands. Lane 1 and 7: genotype 4 (288 bp); Lane 2: negative control; Lane 3: non-typeable; Lane 4: genotype 1a (129 bp); Lane 5: genotype 1b (233 bp); Lane 6: genotype 3a (258 bp); M: DNA ladder (50 bp)

**Figure 3:**
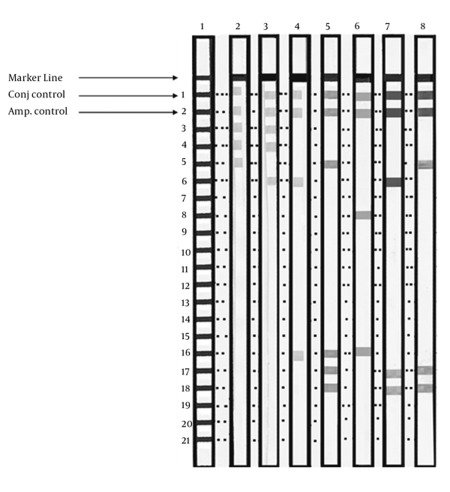
HCV Genotyping Using the INNO-LiPA HCV II Assay System. Lane 1: reference; Lane 2: genotype 1a; Lane 3: genotype 1b; Lane 4: genotype 4a; Lane 5: genotype 4c/d; Lane 6: genotype 4e; Lane 7: genotype 4h; Lane 8: genotype 4

Of the 100 serum samples that tested positive for anti-HCV, 73 (73%) specimens were found to be positive by RT-PCR. There was a predominance of males among HCV RNA-positive patients: 58/73 (79.5%) males versus 15/73 (20.5%) females. Out of 73 RNA-positive samples, 70 (96%) were successfully genotyped by the modified multiplex PCR system; the remaining 3 samples (4%) were nontypeable (all male patients). Of the 70 typeable samples, 55 (79%) were from male patients, while 15 (21%) were from female patients. Typeable samples were distributed as follows: 1 was genotype 1a (1.43%); 1 was genotype 1b (1.43%); 3 were genotype 3a (4.28%); and 65 were genotype 4 (a-h) (92.86%). Figure 2 shows genotype-specific bands byagarose gel electrophoresis. By INNO-LiPA assay, 72/73 (98.6%) samples were successfully genotyped: 57/72 (79%) were males versus 15/72 (21%) females. Only 1 of 73 (1.4%), a male patient, was non-typeable. Typeable samples had the following distribution: 1 was genotype 1a (1.4%); 1 was genotype 1b (1.4%); 3 were genotype 3a (4.2%); 38 were genotype 4 (52.8%); 2 (2.8%) were genotype 4a; 18 were genotype 4c/4d (24.9%); 5 were genotype 4e (6.9%); and 4 were genotype 4h (5.6%). Figure 3 shows a sample of INNO-LiPA HCV II results. Based on a comparison of the results of both assays (Table 2), the genotypes of samples that were typeable by the modified multiplex PCR method were the same as those typed by the INNO-LiPA assay (100% concordance). Of the 3 samples that were not typeable by the first assay, 2 were typeable by the second: 1 was typed genotype 4 and the other was typed 4c/4d. The genotype of 1 sample was non-typeable by either assay. A significant positive correlation was observed between both methods (r = 0.874, P < 0.001). By kappa test, there was excellent agreement (kappa = 0.848) in the results between the two assays.

**Table 2:** Comparison of Results Generated by Both Systems

**HCV Genotype**	**Modified Multiplex PCR Method (Genotype Specific Primers)**		**INNO-LiPA HCV II (Line Probe Assay)**	
	**Isolates, No.**	**Percentage**	**Isolates, No.**	**Percentage**
1a	1	1.37	1	1.37
1b	1	1.37	1	1.37
3a	3	4.11	3	4.11
4	65	89.04	38	52.05
4a	-	-	2	2.74
4c/4d	-	-	18	24.66
4e	-	-	5	6.85
4h	-	-	4	5.48
Not Genotyped	3	4.11	1	1.37
Total	73	100	73	100

## 5. Discussion

There is increasing evidence that patients who are infected with different HCV genotypes have disparate clinical profiles, liver disease severities, and responses to current combination therapy. Hence, a convenient and reliable genotyping system is essential for large-scale epidemiological and clinical studies [18][19]. Although HCV-4 is the cause of approximately 20% of the 170 million cases of chronic hepatitis C worldwide, it has not been the subject of significant research; thus, the features of this genotype and management strategies for patients who have been infected with this genotype are not as well developed as for genotypes 1, 2, and 3 [20]. HCV-4 is a very heterogeneous genotype that displays significant genetic divergence and more subtypes than other genotypes. To date, 18 subtypes have been identified [21][22]. However, the full clinical significance of HCV-4 subtypes is not known, because few studies have been conducted on the correlation between HCV-4 subtypes and the natural history of the disease, pathogenicity, disease severity, and therapeutic outcomes [23][24]. In this study, HCV chronic hepatitis was more frequent in males over females (3.9:1), confirming earlier reports [14][25][26][27][28][29][30]. This observation may be related in part to social risk factors for HCV transmission, such as drug use, schistosomiases, and occupational exposure. However, the role of androgens in the gender bias must not be excluded [14]. In this study, a novel modified multiplex nested PCR method was compared directly with a commercial INNO-LiPA assay method for HCV genotyping. Based on our results, the modified PCR method had a similar level of accuracy as the INNO-LiPA method but was simpler to use and significantly less expensive. In fact, all 70 typeable genotypes obtained by the modified PCR method matched those obtained from the INNO-LiPA method (100% concordance). Two of the 3 samples that were non-typeable by modified multiplex PCR method were typeable by INNO-LiPA method: 1 was typed genotype 4, and the other was 4c/4d. The genotype of 1 sample was non-typeable by either method, likely due to low viral load (r = 0.874, P < 0.001). More importantly, the modified multiplex PCR method was appropriate for typing the prevalent HCV strains in our patient population. In fact, the HCV genotype 4 was detected in 65 out of 70 patients (92.86%), supporting earlier studies [14][31][32][33][34][35][36]. However, our data also show that the contribution of genotype 4 to the study pool is not exclusive and that other genetically related genotypes exist in the population, confirming previous studies [14][37][38]. One cansuggest a deficiency of the multiplex PCR in subtyping genotype 4, but inclusion of more primers would be an unjustified burden on an already financially challenged health care system, in the absence of sufficient evidence to support clinical and therapeutic outcomes [23][24].

One limitation of this multiplex method is the rarity of HCV genotypes other than genotype 4 in Egypt. However, in the original work by Idrees, 2008, the most prevalent genotype in Pakistan (3a) was detected with high frequency. Lower frequencies of other genotypes (3b,1a,3c,1b,2a,4,1c) were also observed. He suggested that due to higher sensitivity of the method, it may be useful for detecting other genotypes in regions of the world where they are predominant to validate its suitability for these genotypes (eg, 5a and 6a) [15]. In conclusion, genotype 4 was the most prevalent genotype in our study. Based on our results, the new, modified multiplex nested PCR assay that we have presented is a sensitive, inexpensive alternative for HCV genotyping and is capable of reliably genotyping HCV RNA directly from clinical samples; thus, this novel assay can be used in routine diagnostic laboratories. Furthermore, INNO-LiPA may be useful as a second-line assay for genotyping samples that are indeterminate by multiplex PCR. This combination will lead to better patient evaluation, better treatment optimization, and a reduction in the spread of HCV. Moreover, the use of both genotyping assays in Egyptian patients might be valuable for large-scale genotyping projects on a national level.
